# Supplementary figures and images for: Deep Learning Modeling of Androgen Receptor Responses to Prostate Cancer Therapies
Source: Int J Mol Sci. 2020 Aug 14;21(16):5847. doi: 10.3390/ijms21165847 (PMC7461580; doi:10.3390/ijms21165847)

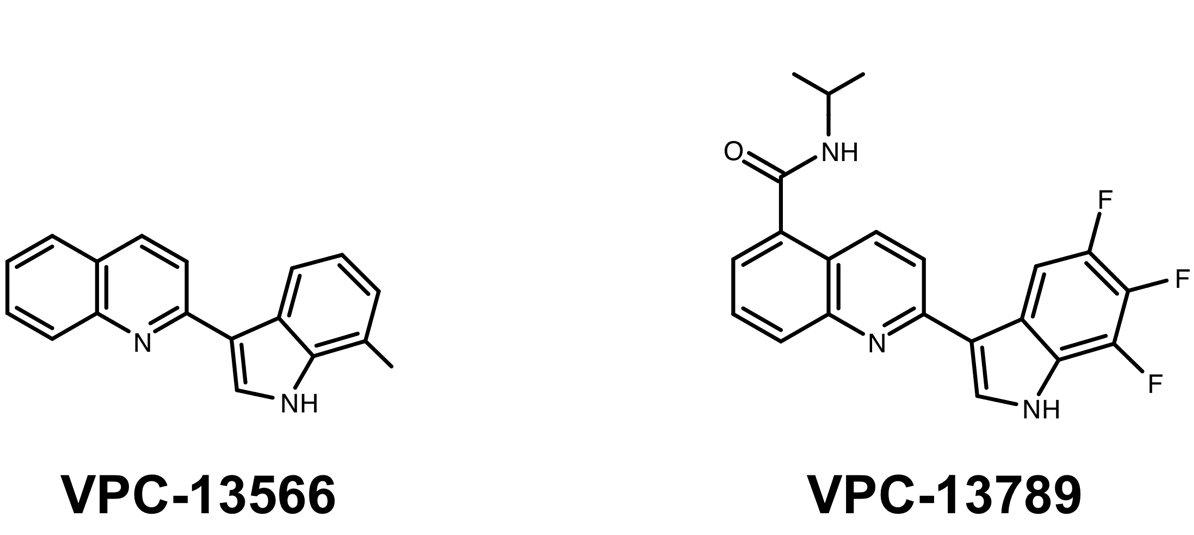

Supplement: Supplementary file 1 [file ijms-21-05847-s001.zip › VPC_antiandrogens.png]

SVM precision vs. recall curve for each class

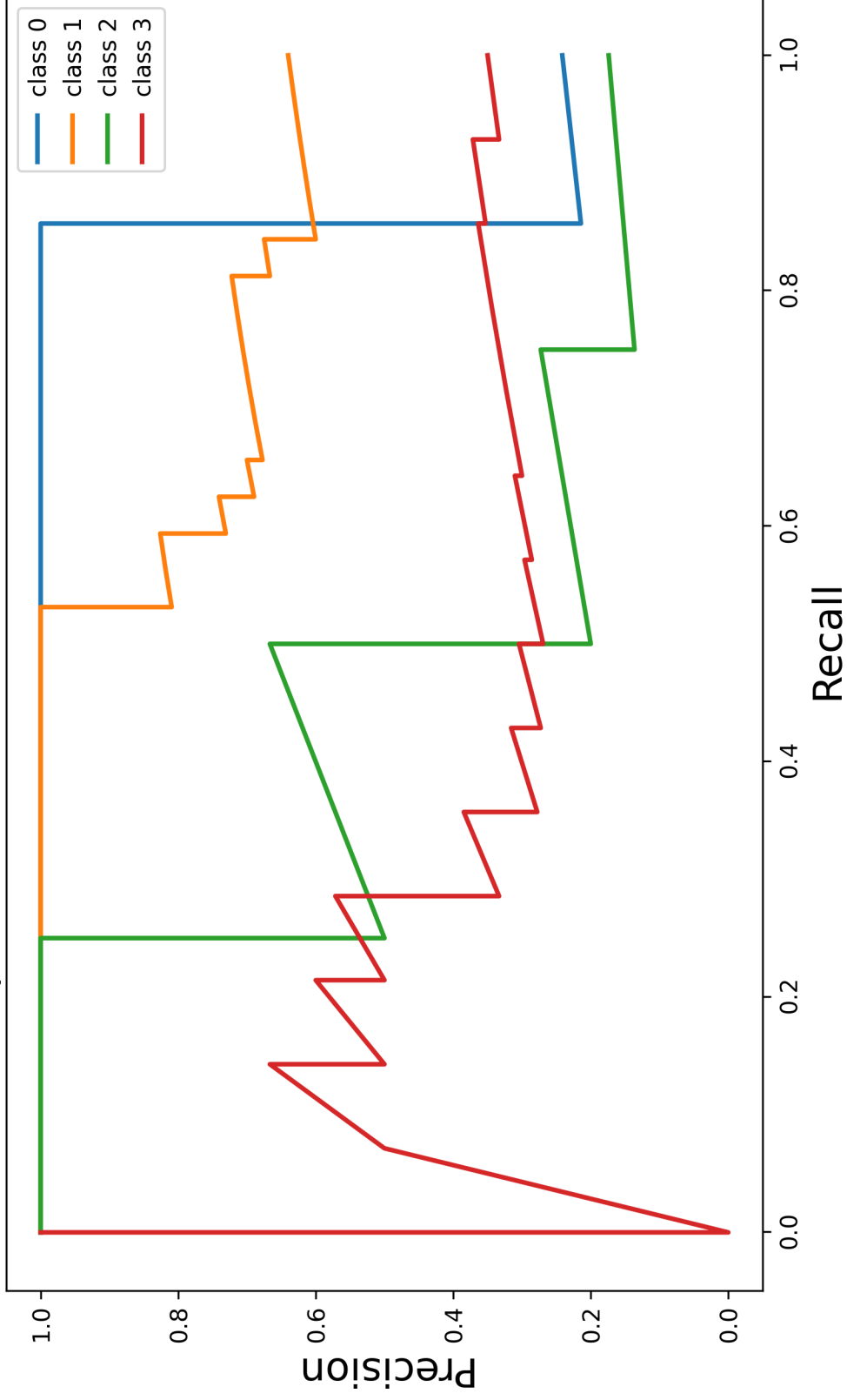

Supplement: Supplementary file 1 [file ijms-21-05847-s001.zip › Precision_recall_curves.pdf]
